# Development and validation of a logistic regression-based nomogram for predicting 1-year all-cause death and heart failure rehospitalization in hypertensive patients with chronic heart failure

**DOI:** 10.3389/fcvm.2026.1877085

**Published:** 2026-08-12

**Authors:** Suqin Wang, Hongzhi Liu

**Affiliations:** Department of Heart Failure, Fuwai Central China Cardiovascular Hospital, Zhengzhou, Henan, China

**Keywords:** hypertensive heart disease, platelet-to-highdensity lipoprotein cholesterol ratio, nomogram, prediction model, prognosis

## Abstract

**Objective:**

Patients with hypertension and chronic heart failure (CHF) face high risks of poor prognostic outcomes. Effective prevention and management strategies remain a priority. However, the predictive value of platelet-to-high-density lipoprotein cholesterol ratio (PHR) for poor clinical outcomes is unclear. This study aimed to develop and internally validate a predictive nomogram incorporating PHR for assessing the risk of composite endpoint of all-cause death and rehospitalization due to HF in hypertensive CHF patients.

**Methods:**

One hundred eighty-three hypertensive CHF patients admitted to Fuwai Central China Cardiovascular Hospital in 2021 were classified into Event-group (*n* = 83) and non-Event group (*n* = 100), with a 12-month follow-up for poor clinical outcomes (all-cause death and heart failure rehospitalization). The composite endpoint (all-cause death or HF rehospitalization) occurred in 83 patients, yielding an overall cumulative incidence of 45.36%. Independent predictors were identified by multivariate logistic regression, and a nomogram was developed and validated by bootstrap resampling. Model performance was assessed using receiver operating characteristic (ROC) curves, calibration charts, and decision curve analysis.

**Results:**

History of atrial fibrillation (AF), increased left atrial diameter (LAD), higher neutrophil-to-lymphocyte ratio (NLR), and elevated PHR are independent risk factors for poor clinical outcomes (all *P* < 0.05). The nomogram incorporating these factors showed strong discrimination with an AUC of 0.814 (95% CI: 0.750–0.878; Hosmer–Lemeshow *χ*^2^ = 4.0329, *P* = 0.2579) in the modeling group and 0.809 (95% CI: 0.746–0.872; Hosmer–Lemeshow *χ*^2^ = 2.5868, *P* = 0.4598) in the validation group. Decision curve analysis confirmed clinical utility across broad threshold probability ranges (modeling group: 12%–82%, validation group: 10%–84%).

**Conclusions:**

The nomogram incorporating PHR effectively predicts all-cause death and heart failure rehospitalization risk in hypertensive CHF patients, showing robust clinical applicability.

## Introduction

Hypertension ranks among the most prevalent chronic cardiovascular diseases (CVDs), affecting nearly 1.39 billion adults globally (1), and constitutes a primary risk factor for CHF. Long-term uncontrolled hypertension triggers progressive cardiac structural and functional remodeling, including left ventricular hypertrophy and left atrial enlargement, ultimately increasing HF risk (2). Hypertensive patients with concomitant HF face substantially elevated risks of adverse cardiovascular events, making risk stratification a key public health priority (3).

Chronic inflammation participates in the onset and progression of hypertension and hypertensive target organ damage; reliable inflammatory biomarkers help optimize risk evaluation for this population (4, 5). The neutrophil-to-lymphocyte ratio (NLR), a widely used inflammatory marker, has been confirmed to independently predict long-term all-cause mortality among patients with heart failure (6).

Platelets and high-density lipoprotein cholesterol (HDL-C) are also closely involved in cardiovascular inflammatory and metabolic regulation, with confirmed associations with hypertension and HF progression (7–9). Nevertheless, the prognostic value of single HDL-C or platelet parameters remains controversial in HF populations, with inconsistent results across previous studies (10). Recent literature indicates that the platelet-to-HDL-C ratio (PHR), serving as an emerging biomarker reflective of inflammatory and metabolic status, offers robust diagnostic and predictive utility in diseases such as non-alcoholic liver disease, chronic kidney disease, and newly identified metabolic syndrome (11). Elevated PHR levels have also been recently linked with heightened risks of hypertension, heart failure, and cardiometabolic multimorbidity (CMM) among middle-aged and elderly populations with hypertension (8, 11, 12). Additionally, PHR has shown a positive and linear correlation with cardiovascular mortality among stroke survivors (13). However, no clinical study has explored the association between PHR and long-term adverse outcomes in hypertensive patients with CHF. Whether PHR can serve as a reliable prognostic indicator for all-cause death and HF rehospitalization in this specific population remains unknown.

Given the above research gaps, this study constructed and internally validated a novel logistic regression-based nomogram incorporating PHR to predict the 1-year risk of all-cause death and HF rehospitalization in hypertensive CHF patients. We aimed to provide a simple, accurate, and clinically feasible prediction tool for individualized prognostic assessment and targeted intervention in this high-risk cohort.

## Materials and methods

### Patient selection and study design

The study enrolled 183 hypertensive patients with CHF admitted from January to December 2021 at the Heart Failure Ward, Fuwai Central China Cardiovascular Hospital. Criteria for participant inclusion encompassed: (1) age over 18 years; (2) documented diagnosis of primary hypertension; and (3) CHF confirmed according to the 2016 European Society of Cardiology guidelines. Patients were excluded if they had: (1) acute myocardial infarction; (2) valvular heart diseases, rheumatic cardiac conditions, or cardiomyopathies of restrictive or hypertrophic nature; (3) severe hepatic or renal dysfunction; (4) significant infections; (5) severe anemia; or (6) hematological conditions, autoimmune diseases, or malignancies. The study was conducted in accordance with the Declaration of Helsinki, and the protocol was approved by the Ethics Committee of Fuwai Central China Cardiovascular Hospital (No. 81 of 2024).

### Clinical data collection

Collected clinical parameters included demographic information such as age and gender, alongside medical history details including AF, diabetes mellitus, coronary artery disease, and laboratory measurements such as N-terminal pro-brain natriuretic peptide (NT-proBNP), uric acid (UA), and creatinine (Cr). Laboratory tests were performed by collecting peripheral venous blood at admission and the following morning under fasting conditions. NT-proBNP was measured by electrochemiluminescence using a Roche Cobas e602 analyzer. Echocardiographic parameters were measured by an experienced sonographer using a GEE95 diagnostic ultrasound system (GE, USA).

### Inflammatory markers

The platelet-to-HDL-C ratio (PHR) was computed as platelet count (×10⁹/L) divided by high-density lipoprotein cholesterol (HDL-C, mmol/L). Monocyte-to-HDL-C ratio (MHR), neutrophil-to-HDL-C ratio (NHR), and uric acid-to-HDL-C ratio (UHR) were calculated via the same equation. Notably, both uric acid (UA) and HDL-C were converted to mg/dL when computing UHR.

UHR=UA(mg/dl)/HDL−C(mg/dl)

For the purpose of this study, we custom-defined three additional ratios: MLR, NLR and PLR, which were calculated as monocyte count, neutrophil count and platelet count divided by HDL-C respectively, following the PHR calculation protocol.

### Clinical outcomes

Patients underwent a 12-month follow-up post-discharge via outpatient visits or telephone interviews. The occurrence of all cause death or hospital readmission due to heart failure defined a composite endpoint, terminating follow-up upon event occurrence. Subsequently, patients were stratified into Event and non-Event cohorts accordingly.

### Statistical analysis

Statistical analyses were conducted with R software (version 4.2.1). Continuous variables were reported as mean ± standard deviation (SD) or median with interquartile ranges (Q1, Q3). Differences between groups were tested using independent-sample t-tests or Mann–Whitney U tests, as appropriate. Categorical data were expressed as percentages and analyzed using the chi-square test.

Variables yielding a *P* < 0.1 in univariate analysis or those clinically important were incorporated into the multivariate logistic regression model. A rigid univariate screening threshold of *P* < 0.05 may exclude variables with borderline univariate significance yet independent predictive effects in multivariate analyses, particularly with a limited sample size. Therefore, we followed the standard two-step variable selection for clinical prediction models: candidate predictors were first retained with a loose univariate cutoff of *P* < 0.1, then backward stepwise logistic regression with a strict entry criterion of *P* < 0.05 was used to identify independent risk factors. This method is supported by methodological basis (14) and a published nomogram study in reproductive medicine to support the threshold of *P* < 0.1 for univariate screening (15).

Before multivariate logistic regression modeling, variance inflation factor (VIF) was calculated to detect multicollinearity among the four included variables. A commonly used cutoff VIF < 10 indicates no obvious multicollinearity. VIF < 5 denotes negligible collinearity. The VIF values of all four predictors in our model were less than 5, confirming that severe multicollinearity did not exist between covariates, and the regression model was statistically robust.

Independent predictors were identified via multivariate logistic regression employing a backward stepwise regression (LR method) with adjustment for potential confounders. Subsequently, these results were applied to establish the nomogram. The predictive capacity of the nomogram was examined using ROC curves; calibration accuracy was depicted via calibration graphs; and clinical relevance was evaluated using decision curve analyses. Bootstrap resampling, with 1,000 iterations, facilitated internal model validation. Statistical significance was set at a *P* < 0.05.

## Results

### Baseline characteristics

A total of 183 hypertensive patients diagnosed with CHF were recruited, among whom 83 patients developed composite endpoint events (45.36%) during 1-year follow-up. Intergroup analysis revealed significant variations (*P* < 0.05) regarding AF history, LAD, estimated glomerular filtration rate (eGFR), NLR, and PHR. Patients experiencing all-cause mortality and heart failure rehospitalization exhibited a higher prevalence of AF history, increased LAD, elevated NLR and PHR values, and reduced eGFR compared to their counterparts (Table 1).

**Table 1:** Baseline characteristics of patients.

Indicators	Non-event (*n* = 100)	Event (*n* = 83)	*P*
Age (years)	53.41 ± 17.42	56.13 ± 16.11	0.278
Sex(M/F)	66 (66.0%)/34 (34.0%)	61 (73.5%)/22 (26.5%)	0.273
Co-morbidities			
Atrial fibrillation (*n* (%))	17 (18.0%)	31 (37.3%)	0.002^a^
Type 2 diabetes mellitus (*n* (%))	25 (25.0%)	29 (34.9%)	0.142
Coronary heart disease (*n* (%))	27 (27.0%)	20 (24.1%)	0.654
Types of Heart failure (*n* (%))			
HFrEF	41 (41.0%)	41 (49.4%)	0.458
HFmrEF	25 (25.0%)	20 (24.1%)	
HFpEF	34 (34.0%)	22 (26.5%)	
Laboratory tests			
Uric acid	7.41 ± 2.76	6.98 ± 2.34	0.261
HDL-C	36.53 ± 10.06	37.06 ± 9.69	0.720
C-reactive protein	3.34 (1.77,6.44)	3.22 (1.50,7.13)	0.795
RDW	42.61 ± 6.58	43.27 ± 5.71	0.478
NT-proBNP	1180.50 (377.85,2323.25)	1187.00 (460.10,3731.00)	0.579
Creatinine	77.00 (67.25,97.50)	87.00 (70.00,115.00)	0.055
eGFR	76.54 ± 20.58	69.38 ± 21.60	0.023^a^
MLR	0.23 (0.18,0.34)	0.27 (0.19,0.35)	0.205
NLR	2.05 (1.54,3.11)	3.17 (2.26,5.20)	<0.001^a^
PLR	126.44 (94.57,164.25)	120.73 (92.57,150.24)	0.403
MHR	0.48 (0.34,0.64)	0.44 (0.32,0.62)	0.682
NHR	4.84 (3.20,6.21)	4.27 (3.08,5.57)	0.256
PHR	209.31 (154.78,272.41)	254.14 (220.22,338.10)	<0.001^a^
UHR	0.20 (0.15,0.28)	0.19 (0.13,0.25)	0.228
Echocardiographic parameters			
LAD	43.12 ± 4.96	47.23 ± 5.66	<0.001
Ejection fraction	42.91 ± 13.31	42.14 ± 13.10	0.697
LVEDD	58.60 ± 11.59	58.93 ± 10.90	0.845
E/e’	17.17 ± 5.67	16.93 ± 4.73	0.752
TRV	2.55 ± 0.46	2.49 ± 0.47	0.386
PASP	33.00 (25.25,41.75)	29.00 (25.00,42.00)	0.426

HFrEF, heart failure with reduced ejection fraction; HFmrEF, heart failure with mildly reduced ejection fraction; HFpEF, heart failure with preserved ejection fraction; HDL-C, high-density lipoprotein cholesterol; RDW, red blood cell distribution width; NT-proBNP, N-terminal pro-brain natriuretic peptide; eGFR, estimated glomerular filtration rate; LAD, left atrial anteroposterior diameter; LVEDD, left ventricular end-diastolic diameter; E/e’, early trans-mitral flow velocity to early diastolic mitral annular velocity ratio; TRV, tricuspid regurgitation velocity; PASP, pulmonary artery systolic pressure; MLR, monocyte-to-lymphocyte ratio; NLR, neutrophil-to- lymphocyte ratio; PLR, platelet-to-lymphocyte ratio; MHR, monocyte-to-high-density lipoprotein cholesterol ratio; NHR, neutrophil-to-high-density lipoprotein cholesterol ratio; PHR, platelet- to- high-density lipoprotein cholesterol ratio; UHR, uric acid-to-high-density lipoprotein cholesterol ratio.

a Statistically significant difference. Continuous variables are expressed as mean ± standard deviation or medians with the lower and the upper quartile, whereas categorical variables are expressed as (*n*) with a percentage (%).

### Risk factors for all-cause mortality and heart failure rehospitalization in hypertensive patients with CHF

Patients variables identified as statistically significant through univariate analyses or those deemed clinically pertinent were integrated into a multivariate logistic regression model with a selection threshold of *P* < 0.1. To mitigate the potential issue of multicollinearity, backward stepwise regression (LR method) was applied. Results indicated that AF history, LAD, NLR, and PHR independently predicted all-cause mortality and heart failure rehospitalization, achieving statistical significance (*P* < 0.05) (Table 2).

**Table 2:** Multivariable logistic regression analysis of risk factors for all-cause mortality and HF rehospitalization in patients with hypertension and CHF.

Parameter	OR	95% CI	*P*-Values
Atrial fibrillation	3.114	1.374–7.058	0.007
LAD	1.169	1.090–1.255	0.000
NLR	1.349	1.089–1.670	0.006
PHR	1.005	1.001–1.009	0.020
intercept	0.000		0.000

LAD, left atrial anteroposterior diameter; NLR, neutrophil-to-lymphocyte ratio; PHR, platelet-to- high-density lipoprotein cholesterol ratio.

### Construction of the nomogram model

**Figure 1:**
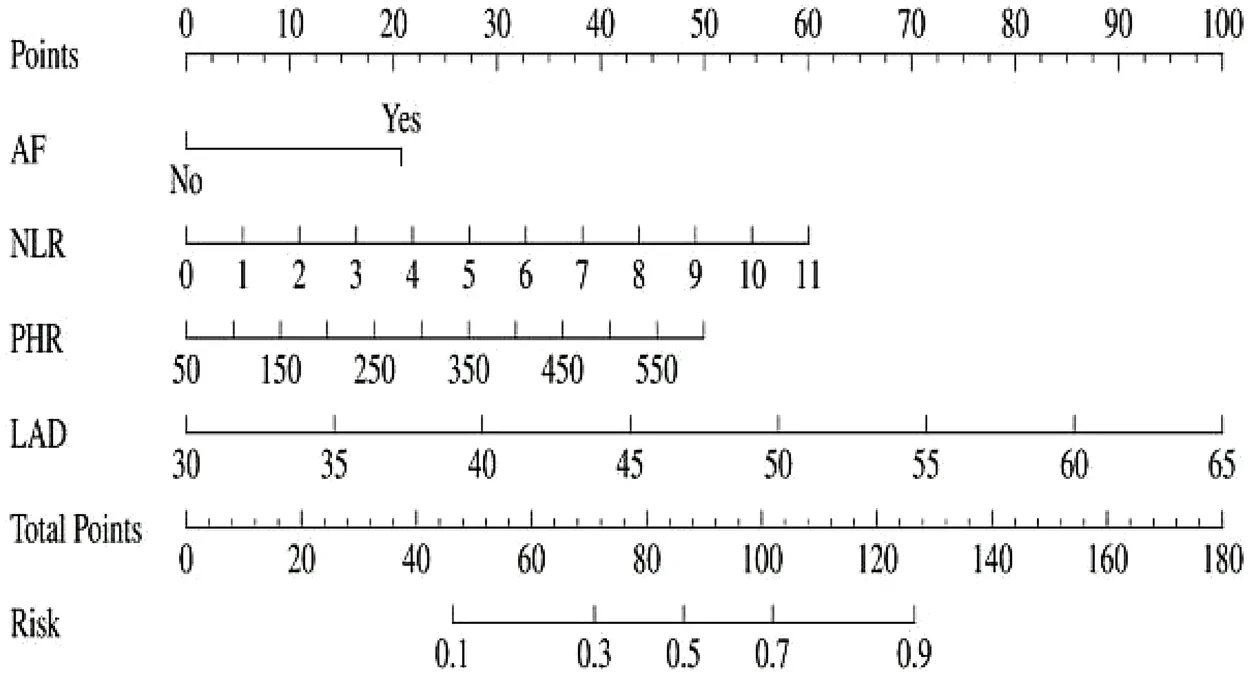
Nomogram model for predicting the risk of all-cause death and HF rehospitalization in hypertensive CHF patients. AF, atrial fibrillation; NLR, neutrophil-to-lymphocyte ratio; PHR, platelet-to-high-density lipoprotein cholesterol ratio; LAD, left atrial anteroposterior diameter.

The four independent predictors established from multivariate logistic regression analysis were employed to develop a predictive model for assessing all-cause mortality and heart failure rehospitalization risk in hypertensive CHF patients, visually represented as a nomogram (Figure 1). In this nomogram, each predictor corresponds to a score. The individual's total score, calculated by summing each predictor's assigned value, reflects the predicted probability of experiencing the composite endpoint of all-cause mortality and heart failure rehospitalization.

### Validation and evaluation of the nomogram model

**Figure 2:**
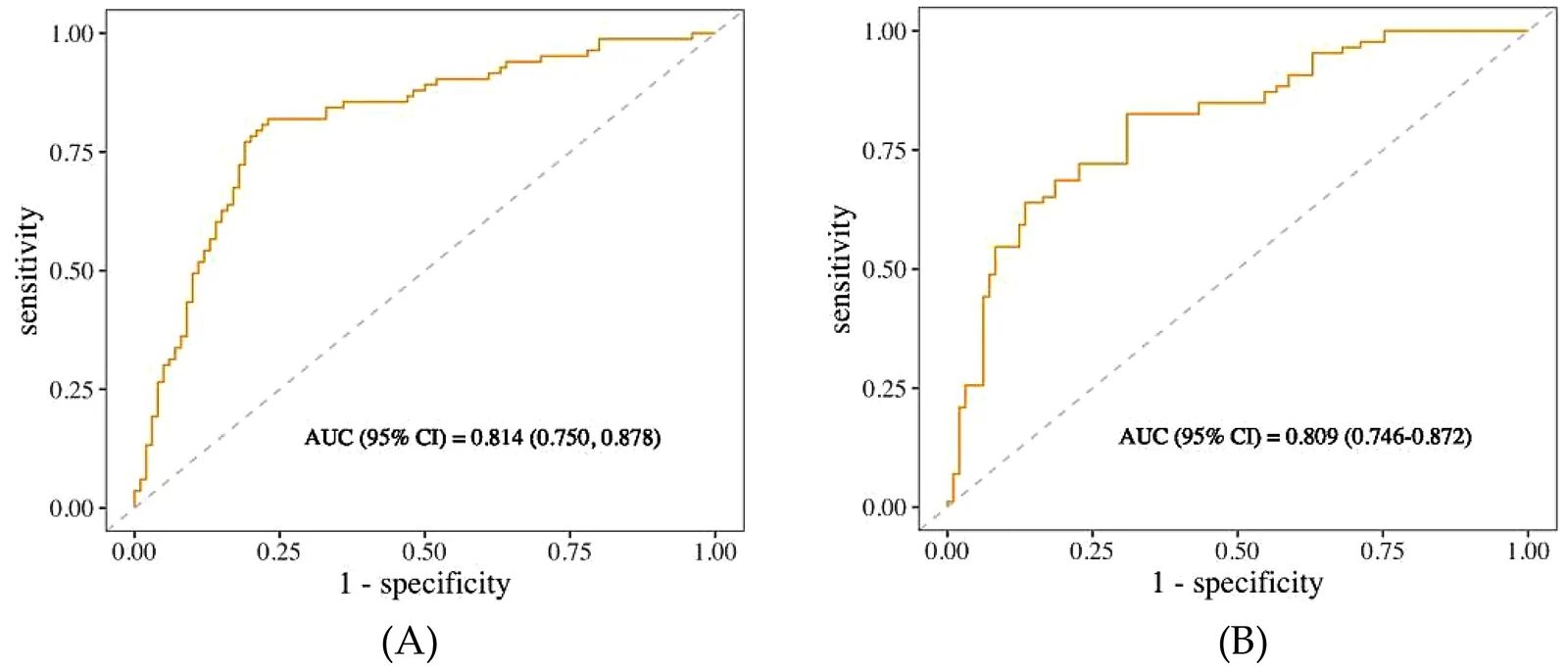
ROC curves of nomogram model for prediction of of all-cause death and HF rehospitalization in hypertensive patients with CHF. **(A)** The modeling group; **(B)** the invalidation group.

**Figure 3:**
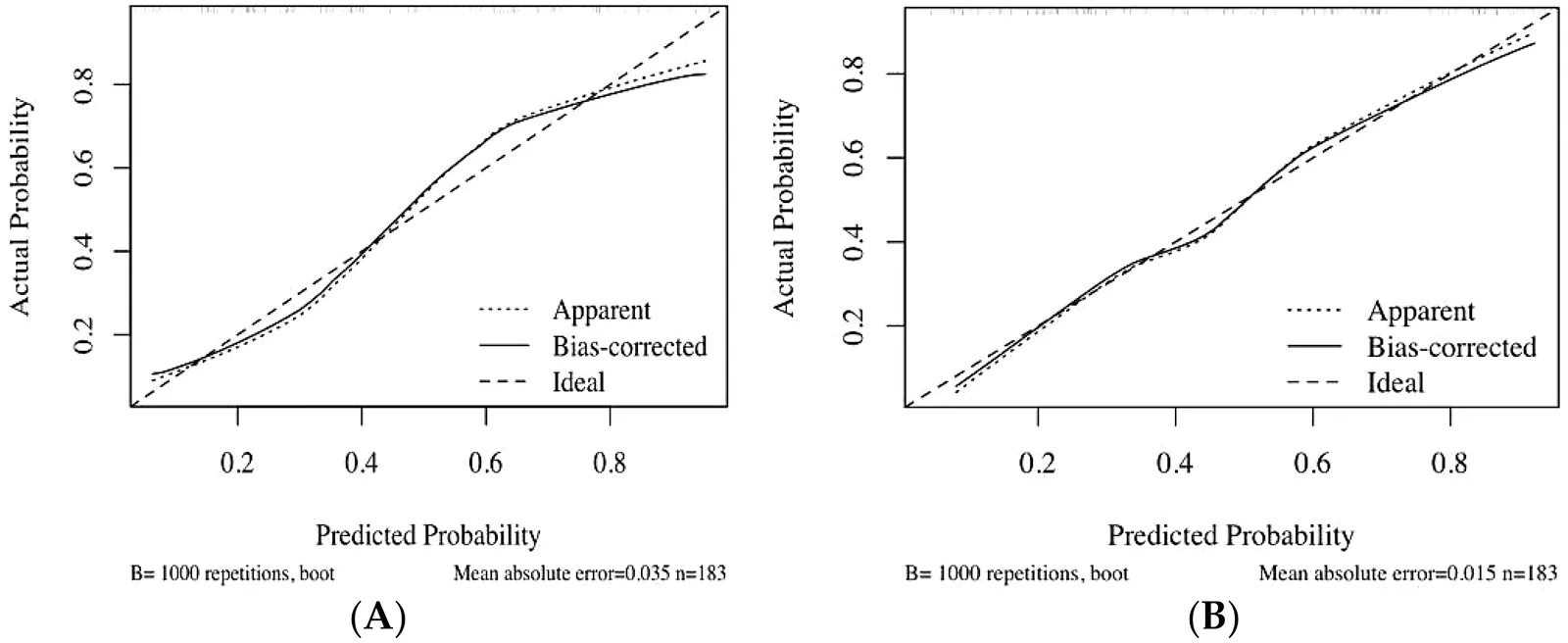
Calibration curves of the nomogram model for predicting all-cause death and HF rehospitalization in hypertensive patients with CHF. **(A)** The modeling group; **(B)** the invalidation group.

**Figure 4:**
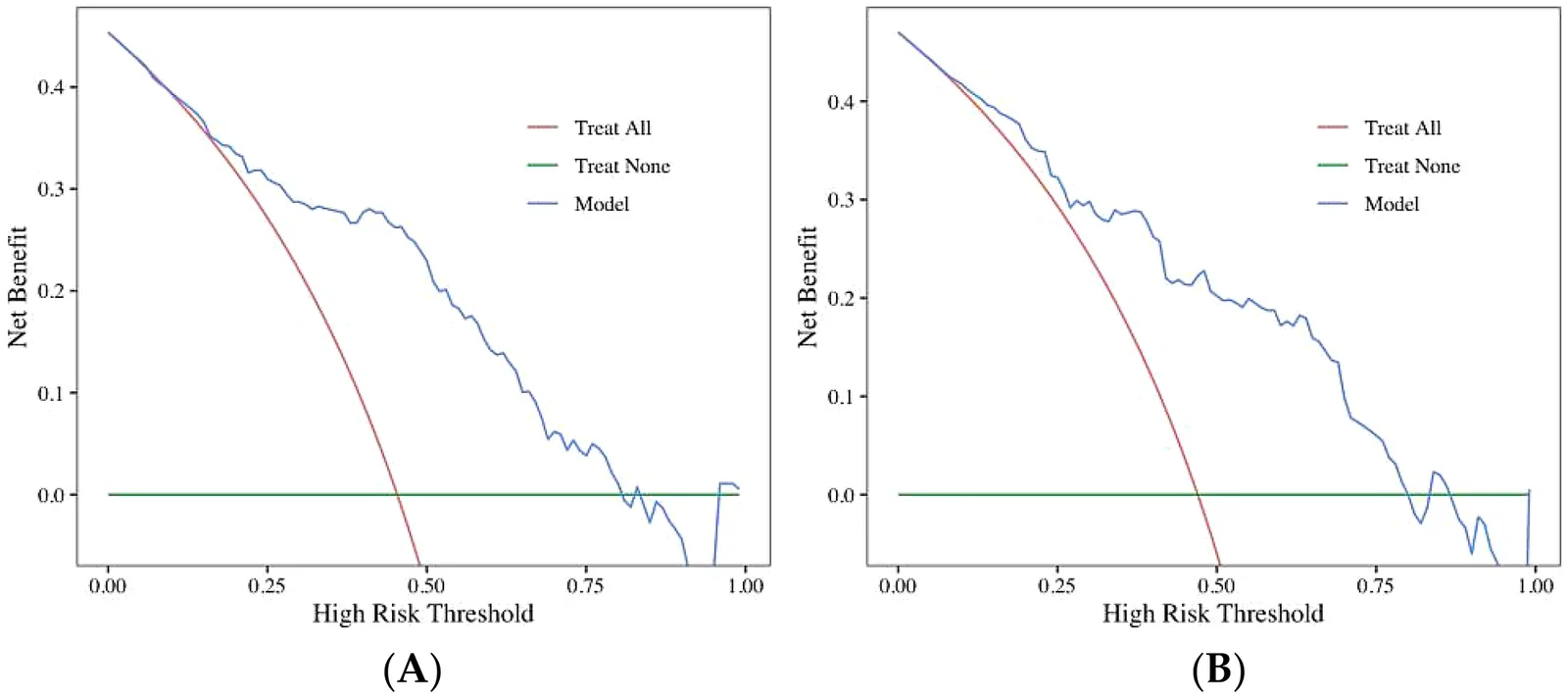
Decision curves of the nomogram model for predicting all-cause death and HF rehospitalization in hypertensive patients with CHF. **(A)** The modeling group; **(B)** the invalidation group.

The ROC curve assessment revealed an area under the curve (AUC) of 0.814 (95% CI: 0.750–0.878) for the modeling group, with a cutoff value of 0.424, showing sensitivity and specificity values of 0.819 and 0.770, respectively. Internal verification via 1,000 cycles of bootstrap resampling yielded an AUC of 0.809 (95% CI: 0.746–0.872) in the validation set, establishing an optimal threshold at 0.378, specificity at 0.826, and sensitivity at 0.691, thus affirming the model's strong discriminative performance (Figure 2). Calibration evaluation exhibited satisfactory consistency between the predicted and actual outcomes, presenting Hosmer–Lemeshow *χ*^2^ values of 4.0329 (*P* = 0.2579) for the modeling group and 2.5868 (*P* = 0.4598) for the validation group (Figure 3). Decision curve evaluation highlighted significant net clinical benefits within probability thresholds from 12% to 82% for the modeling group and 10% to 84% for the validation group, validating the clinical effectiveness of the model (Figure 4).

## Discussion

This study enrolled individuals admitted with CHF accompanied by hypertension, using all-cause mortality and rehospitalization due to heart failure within one year following discharge as the primary outcome. The study comprehensively assessed 26 variables, including clinical indicators, echocardiographic measures, and routine inflammatory biomarkers. A personalized risk nomogram was constructed utilizing multivariate logistic regression analysis. The findings demonstrated that higher PHR and NLR levels, atrial fibrillation history, and enlarged LAD independently served as predictors of composite endpoint of all-cause mortality and heart failure rehospitalization in this group. The nomogram, incorporating these predictors, demonstrated an AUC of 0.814 within the modeling cohort and 0.809 upon internal verification. Calibration plots illustrated robust predictive accuracy, while decision curve analysis indicated noteworthy clinical net advantages, highlighting the reliability and practical utility of the developed nomogram. This study developed a simple and intuitive tool for assessing one-year adverse outcome risk in hypertensive patients with CHF, providing a reliable basis for precise risk stratification before discharge.

Multiple studies confirmed that PHR, as a novel biomarker, significantly correlates with hyperuricemia, and targeted interventions on PHR may reduce hyperuricemia risk (16). Additional studies revealed a close association between PHR and increased CVD mortality risk in patients with depression (17). Patients with CMM have become a clinical priority due to their higher mortality risks. Research showed hypertensive patients with cardiovascular comorbidities face a 2.36-fold increase in all-cause mortality risk (18). Thus, accurately identifying high-risk CMM populations and initiating early interventions are crucial for improving survival. Our analysis indicated that elevated PHR independently correlated with all-cause death and heart failure readmission risk in hypertensive patients with CHF, aligning with previous findings. This further validates the prognostic value of PHR in CVD, supporting its use in clinical early interventions and adverse event prevention among hypertensive patients with CHF and elevated PHR.

The pathophysiological mechanisms underlying hypertension and heart failure primarily involve neuroendocrine activation and chronic inflammation (19, 20), in which platelets play an essential role (21). Studies indicated that decreased platelet counts correlate with increased all-cause mortality and heart failure rehospitalization risks in heart failure patients (22). However, other studies suggested that elevated platelet counts might indicate poor cardiovascular outcomes (23), and these findings remain controversial. HDL-C possesses anti-inflammatory and antioxidant properties (24), However, its association with cardiovascular outcomes varies across populations: in stroke patients, HDL-C showed no significant relationship with cardiovascular mortality (13) in hypertensive males, a U-shaped association exists between HDL-C and cardiovascular event risk (9), whereas in heart failure patients, some studies reported no significant association or even negative correlations between HDL-C levels and outcomes (8, 10). PHR combines platelet count and HDL-C levels, with elevated values simultaneously reflecting increased platelet counts and decreased HDL-C levels. This study demonstrated that HDL-C alone showed no significant correlation with adverse outcomes among hypertensive CHF patients. Notably, increased PHR independently predicted all-cause mortality and heart failure rehospitalization, suggesting superior prognostic utility compared to individual indicators alone in this population.

Abnormal peripheral blood neutrophil and lymphocyte counts frequently appear in heart failure patients (23). Previous studies have consistently linked alterations in neutrophil and lymphocyte counts to the incidence, severity, rehospitalization rates, and adverse outcomes of heart failure (22, 25–27). The neutrophil-to-lymphocyte ratio (NLR), an inflammatory biomarker combining both cell counts, strongly correlates with cardiovascular outcomes and prognosis (6). A meta-analysis encompassing 25 studies found markedly higher NLR in deceased patients relative to survivors (28); Another meta-analysis involving patients with heart failure and preserved ejection fraction (HFpEF) reported that higher NLR significantly correlated with increased risks of all-cause and cardiovascular death (29). This study observed significantly higher NLR in hypertensive CHF patients who experienced all-cause death and heart failure rehospitalization within one year post-discharge, consistent with prior research. This finding further confirms the clinical utility of NLR as a prognostic predictor for adverse outcomes in this population.

Prolonged hypertension can induce left atrial enlargement and remodeling, resulting in impaired atrial systolic and diastolic functions and further worsening pressure overload. Left atrial enlargement represents a crucial manifestation of cardiac remodeling and may serve as an early marker of hypertensive heart disease in patients with essential hypertension (30). Enlargement and remodeling of the left atrium facilitate the development of AF, which, in turn, further exacerbates left atrial remodeling, creating a vicious cycle (31). Clinically, hypertensive patients with heart failure often exhibit significant left atrial enlargement and are susceptible to concurrent AF, aggravating clinical symptoms and increasing the risk of adverse outcomes. The left atrial diameter (LAD), a critical indicator reflecting cardiac structure and function, possesses significant prognostic value in evaluating patients with hypertension and heart failure. Research also supports a strong association between left atrial enlargement severity and cardiovascular mortality (32). This study confirmed increased LAD and a history of AF as independent predictors for adverse outcomes in hypertensive CHF patients, underscoring the clinical importance of blood pressure management to minimize left atrial remodeling, thus reducing AF incidence and adverse events. Although LAD measurement is comparatively less precise than assessing left atrial volume, it remains an accessible and practical marker for early identification of high-risk patients. Nevertheless, incomplete clinical datasets precluded the calculation of left atrial volume indices, limiting precise evaluation of left atrial dimensions in the current study.

This research has significant clinical translational value. Firstly, inflammatory markers such as PHR can be easily obtained through routine biochemical tests upon admission, eliminating the need for additional testing costs and enhancing practicality, especially in primary healthcare settings with limited resources. Secondly, left atrial enlargement and AF can be effectively controlled through pharmacological or minimally invasive interventions, providing clear targets for clinical management. In the future, the predictive nomogram established in this research could potentially be adapted into a mobile-based application, enabling real-time risk assessment at the bedside and significantly improving clinical practicality.

Through a cross-sectional design, this study first identified a noteworthy correlation between PHR and the occurrence of all-cause mortality and heart failure-related rehospitalization within one year after discharge among hypertensive CHF patients. The findings reinforce the predictive capability of PHR regarding all-cause mortality and heart failure rehospitalization risk within this patient group. Additionally, by employing multivariate logistic regression and subsequently constructing and internally validating a nomogram, the robustness of the link between elevated PHR and adverse outcomes was enhanced, providing novel insights into early preventive measures and risk-stratified hypertension management strategies. However, certain limitations should be acknowledged. Firstly, the single-center, retrospective nature and modest sample size may lead to potential selection biases. Secondly, the current study did not separately analyze individual outcomes such as all cause death and rehospitalization due to heart failure, thus limiting the ability to discern the predictive accuracy of the model for distinct endpoints. Furthermore, the absence of external validation potentially limits the generalizability and precision of the findings. Finally, the relatively short one-year follow-up period restricts the evaluation of longer-term outcomes and mortality. Future research should consider implementing prospective multicenter studies with expanded sample sizes, extended follow-up durations, and external validation procedures. These enhancements would substantially reinforce the model's validity and broaden its clinical relevance, thus offering robust support for tailored clinical management practices.

## Conclusions

This study successfully constructed and internally validated a nomogram prediction model integrating inflammatory biomarkers (PHR, NLR) and clinical indicators (history of AF, LAD) for one-year composite poor prognosis prediction in hypertensive patients with CHF. The model showed good discriminatory ability in the training set (AUC = 0.814) and in the validation set (AUC = 0.809), with acceptable calibration in the modeling group (Hosmer–Lemeshow test *P* = 0.2579) and in the validation group (Hosmer–Lemeshow test *P* = 0.4598). Decision curve evaluation also highlighted significant net clinical benefits.

## Data Availability

The raw data supporting the conclusions of this article will be made available by the authors, without undue reservation.
